# Identifying rare and common variants with Bayesian variable selection

**DOI:** 10.1186/s12919-016-0059-0

**Published:** 2016-10-18

**Authors:** Cheongeun Oh

**Affiliations:** Biostatistics, Department of Population Health, New York University, New York, NY 10016 USA

## Abstract

**Background:**

Recent advances in next-generation sequencing technologies have made it possible to generate large amounts of sequence data with rare variants in a cost-effective way. Yet, the statistical aspect of testing disease association of rare variants is quite challenging as the typical assumptions fail to hold owing to low minor allele frequency (<0.5 or 1 %).

**Methods:**

I present a Bayesian variable selection approach to detect associations with both rare and common genetic variants for quantitative traits simultaneously. In my model, I frame the problem of identifying disease-associated variants as a problem of variable selection in a sparse space, that is, how best to model the relationship between phenotypes and a set of genetic variants. By constructing a risk index score for a group of rare variants, my method can effectively consider all variants in a multivariate model. I also use a within-chain permutation to generate the empirical thresholds to detect true-positive variants.

**Results:**

I apply our method to study the association between increases in baseline systolic and diastolic blood pressure (SBP and DBP, respectively) and genetic variants in the data from Genetic Analysis Workshop 19 unrelated samples. I identify several rare and common variants in the gene *MAP4* that are potentially associated with SBP and DBP.

**Conclusions:**

The application shows that my method is powerful in identifying disease-associated variants even with the extreme rarity.

## Background

With the advent of next-generation sequencing, rare variants with a minor allele frequency (MAF) of less than 1 to approximately 5 % are getting more attention in genome-wide association studies (GWAS) to account for the “missing” heritability phenomenon [1]. Despite the importance, testing for associations between rare variants and disease traits has proven challenging because evaluating the potential impact of rare variants on disease is complicated by their uncommon nature of the extreme rarity. Over the last few years, numerous methods have been developed to address methodological challenges in rare-variant association analysis. Noticeably, multimarker approaches have drawn much attention. Commonly used methods include the collapsing, simple-sum, and weighted-sum methods [2–5]. They first collapse rare variants and then implement a LASSO (least absolute shrinkage and selection operator) [6–8], partial least squares regression (PLS) model [9], or other supporting statistical methods using the common variants and the collapsed rare variants [10].

Although they offer a new way of looking at rare variants, simply pooling these variants may cancel the true signal and, consequently, discard the possibility that multiple rare variants affect phenotype in a different direction of being disease-promoter or disease-protective. Because there is no clear cutoff distinguishing rare variants from common variants, statistical methods that can analyze both rare and common variants simultaneously are often preferable [5, 11, 12]. In this sense, variant association tests can be best approached as a variable selection problem when the main goal is to identify causal variants [12–14]. There has been a parallel development of new statistical methods for detecting rare variants in the Bayesian variable selection framework [12, 15–18].

In the present study, I extend my previous study [19] to the realm of rare variants in a Bayesian variable selection by incorporating a Bayesian risk index approach [16, 17]. By using a risk index score on a group of rare variants over the genomic region, I evaluate both rare and common variants simultaneously. Inference of identifying disease-associated variants is done by estimating marginal posterior probabilities of latent variables. I further perform the within-chain permutation [18] by adopting the idea of permuting the phenotype data in determining the empirical thresholds with regard to true and false signals.

## Methods

### Data

I apply my method to Genetics Analysis Workshop (GAW) 19 unrelated data (*n* = 1934) that were carried out as part of the T2days-GENES (Type 2 Diabetes Genetic Exploration by Next-generation sequencing in multi-Ethnic Samples) consortium to study the association between blood pressure phenotypes and the single-nucleotide polymorphisms. The phenotypes of interest are real baseline diastolic blood pressure (DBP) and systolic blood pressure (SBP), which I consider as continuous traits.

### Model formulation

Suppose that a population-based association study consists of *n* unrelated individuals. Let *Y =* (*Y*_*1*_ *,…, Y*_*n*_)^*T*^ denote the clinical quantitative outcome or response of interest from *n* samples and *X* denote the *n × p*-dimensional genotype matrix of *p* variants in functional genomic regions. Throughout this article, I assume that genetic variants are independent and present no interaction effect. The model I posit on the clinical outcome is

1 $$ Y = {\alpha}_0 + X\beta + \varepsilon $$

where *X* is associated with the coefficients *β.* The error terms *ε* = (*ε*_*1*_ *,…, ε*_*n*_) are assumed to be independent and identically distributed *N*(*0, σ* ^*2*^ *I*_*n*_) for the gaussian responses. I assume an additive genetic model; thus *X*_*ij*_ = 0, 1, or 2, representing the number of minor alleles present at variant *j* of individual *i*. Note that additive model and dominant model are almost equivalent in rare variant analysis.

Given this information, I use Bayesian model uncertainty techniques in which an individual model is specified by the *p*-dimensional vector of binary indicators *γ* = (*γ*_*1*_ *,…, γ*_*p*_). Each component *γ*_*j*_ = 1(0) indicates the inclusion (exclusion) of variant *j*. With a prespecified MAF threshold of defining rare variants, I further assume that the genotype matrix *X* is known to be partitioned into *G* groups such as *X* = (*X*_*1*_ *,…, X*_*G*_), where the *g* ^*th*^ group, *X*_*g*_ contains *k*_*g*_ rare variants (or 1 common variant) for *g = 1,…, G*. Then I define the risk index score as a linear function within the group as *G*_*g*_ *= X*_*g*_ *γ_*g*_, where *G*_*g*_ is a vector of length *n* that gives the risk index for each individual and *γ*_*g*_ contains a vector of binary indicators for a group of *k*_*g*_ rare variants. Then *X* = (*X*_*1*_ *,…, X*_*G*_) becomes *n × G*-dimensional risk index matrix (*G* < *p*). Equation (1) is rewritten in relating the quantitative outcome variable to the risk index matrix by fitting the model *Y = β*_*0*_ *+ Gβ + ε*, where *β* = (*β*_*1*_ *,…, β*_*G*_) is a vector of group-specific coefficients*.*

### Model likelihood and priors

Under the Bayesian method of estimation, computing the degree to which any model represented by *γ* in the model space *M* is supported by the data is calculated via posterior model probabilities of the form $p\left(Y\left|\gamma \right.\right)=\frac{p\left(\gamma \right)p\left(Y\left|\gamma \right.\right)}{{\displaystyle {\sum}_{\gamma \in M}p\left(\gamma \right)p\left(Y\left|\gamma \right.\right)}}$where the nominator entails the multiplication of prior and likelihood function by Bayes’ rule. To calculate the marginal likelihood, I integrate out any dependency on the parameters *β* and *σ*, and use the following approximation in the likelihood function:

$$ p\left(Y\left|\gamma \right.\right)={\displaystyle \iint f\left(Y\left|\beta, {\sigma}^2,\gamma \right.\right)}\;f\left(\beta, {\sigma}^2\right)\cdot d\kern0.2em \beta \cdot d\kern0.1em {\sigma}^2\approx p\left(Y\left|\gamma, \widehat{\beta,}\widehat{\sigma^2}\right.\right) $$

This approximation corresponds to assuming that all of the prior mass of the model-specific parameters, *θ*_*-γ*_ = (*β*_*0*_ *, β, σ*), is placed on the maximum likelihood estimate (MLE). Given the approximation to the marginal likelihood, I am left to define the prior distribution *p*(*γ*) on the model space *M*. To do so, I assume that the number of variants included (nonzero components) in a chosen model *γ* is distributed as a binomial with *π* the prior inclusion probability of each variant, where *π* controls the average number of variants included in the model. Hereafter *π* is referred to as a prior inclusion probability (PIP). Then

$$ p\left(\gamma \right)={\varPi}_{i=1}^p{\pi}^{\gamma i}{\left(1-\pi \right)}^{1-\gamma i} $$

Conditional upon a variant being included, setting *π* = 0.5 yields the uniform prior across the model space. While this prior seems to be noninformative with respect to the model space, it actually can be quite informative in that PIP can control the sparsity of the model by assuming to be smaller.

### Model search using Markov chain Monte Carlo

Once the priors have been chosen, my key construct of interest is the posterior of the *p*-dimensional vector of binary indicators *γ,* which capture the association between variants and the outcome. Markov chain Monte Carlo (MCMC) can often be used to extract such information by simulating an approximate sample from the posterior distribution. Most popular are the Gibbs sampler (GS) [20, 21] and the Metropolis-Hastings (MH) algorithms [22, 23]. In this study, I use the MH algorithm to draw samples from the posterior probability distribution from the model space similarly as in Quintana et al. [16].

### Marginal posterior quantities

I am interested in answering the question of which variant(s) is(are) most likely to derive the association with phenotype. This question can be answered based on marginal posterior probabilities. The marginal posterior probability (MPP) for any *γ*_*j*_ can be calculated as the sum of the posterior probabilities for every model that includes the variable. This is then used as a measure of the evidence of *j* ^th^ variant for association with phenotype and the ranking of their MPPs as a measure of the relative importance. Variants whose MPPs exceed the PIP are selected as being disease-associated, similarly as in my previous study [19].

### Decision rule based a permutation within Markov chain Monte Carlo

Determining true- and false-positive signals in general remains an open problem within Bayesian analysis. Often Bayes factor [24] is considered to be a preferred decision-making rule because it is free of the analyst’s subjectivity and allows the strength of evidence provided by the data in favor of a hypothesis to be evaluated on the widely used empirical scale [25]. Although Bayes factor is a practical tool in a Bayesian context, it has been argued to be sensitive to prior distributions and often becomes computationally intensive in high-dimensional data. However, the permutation test does provide a data-driven decision rule and is conceptually easier to implement. It has been a universal tool in evaluating the significance across various statistical methods. In this study, I implement a permutation within MCMC by adopting the idea of permuting the phenotype data, which is used to determine empirical thresholds in identifying true-positive variants.

In detail, let *y* = {*y*_*j*_} be the vector of the original phenotypes and y* = {*y*_j_*} be the randomly rearranged (permuted) vector of phenotypes. For each iteration, 2 MCMC runs are conducted, one on *p*(*γ|Y*) and the other on *p*(γ|Y*), to generate the empirical posterior distributions under the null model. After variants are screened from the comparison with the PIP, true-positive variants are further identified by gauging their MPPs against ones calculated from the permutation. That is, variants whose MPPs exceed empirical MPPs are declared to be true positives.

## Results

I focused on the *MAP4* gene in chromosome 3 that was previously reported to be highly associated with blood pressure [26]. The sequenced *MAP4* gene had 409 variant sites. Of these, 324 variants were seen at allele frequencies with 0 %. After eliminating 92 individuals with missing data and singleton variants, 85 variants on 1851 unrelated individuals remained. Log-transformation of the phenotype was performed to fix the skewing of the phenotype distribution.

Any division of genetic variants into “common” and “rare” is arbitrary. Although most association studies often use a MAF threshold of 1 % for differentiating between a polymorphism and a mutation, this may not be the optimal threshold for rare variant analysis [5]. I examined the sensitivity of my method to the classification of variants by varying MAF thresholds from starting at 5 %. Choosing the PIP is straightforward. Because it controls the sparsity of the model, a smaller value provides smaller MPPs. Although estimated MPPs are sensitive to the choice of the PIP, the ranking of MPPs is relatively insensitive (data not shown), which was evaluated in my previous work [19]. I fixed PIP as equaling 0.1, which implies that, in expectation, 1 out of 10 variants are included in the model.

For each MCMC run, the convergence of the search was monitored. To see how stable the final estimates were, multiple MCMC runs were also conducted with different initial values and starting points. Convergence of the estimates was checked by the test of convergence diagnostics proposed by Geweke [27] and monitored using the R package CODA.

The chains seemed to reach their target distribution after 5000 or fewer iterations overall. I discarded the first 5000 iterations as a burn-in period. The chain was thinned by keeping 1 observation out of 10 iterations to reduce correlation until the posterior sample size reached 5000. The total number of iterations was 5000 + 5000 × 10 = 55,000. The remaining samples were used to perform inference.

**Fig. 1:**
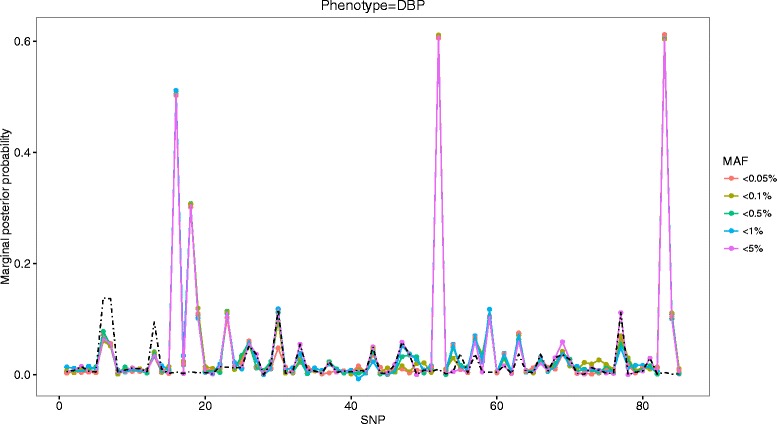
Profiles of MPPs under various MAF thresholds for DBP phenotype in comparison with empirical MPPs (the *black* *dotted-line*)

**Fig. 2:**
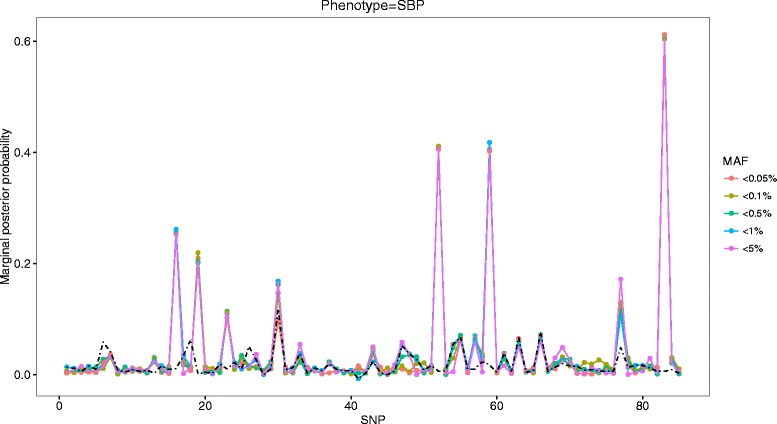
Profiles of MPPs under various MAF thresholds for SBP phenotype in comparison with empirical MPPs (the *black* *dotted-line*)

Figures 1 and 2 depict MPPs for a range of MAF thresholds of defining rarity along with the names of variants written on the *X*-axis that had constant peaks for association with SBP and DBP, respectively. It is clearly shown that candidate variants had strong evidence for association with DBP (3_47956424, 3_48040283, 3_47908815, 3_47912308) and with SBP (3_48040283, 3_47956424, 3_47957996, 3_47908815, 3_47912407, 3_47913606, 3_48016938), consistently exceeding PIP = 0.1, as well as empirical MPPs (the dotted black line) across various MAF thresholds. These remained as being true positives after their averaged MPPs were evaluated against empirical MPPs. On the other hand, among 6 variants whose MPPs were slightly greater than PIP = 0.1, 4 variants (3_47912736, 3_47912407, 3_47957996, 3_48040284) remained to be true positives for association with DBP, whereas the variant (3_47912736) remained to be true positive for association with SBP. The finally selected variants were ordered by their averaged MPPs in Tables 1 and 2.

**Table 1:** True-positive variants in gene *MAP4* ordered by MPPs for association with DBP

Variant	MAF	Averaged MPPs	Empirical MPPs
3_47956424	0.343541	0.6081	0.0136
3_48040283	0.028049	0.6059	0.0115
3_47908815	0.002573	0.5052	0.0049
3_47912308	0.000515	0.3059	0.0152
3_47912736	0.000257	0.1105	0.0099
3_47912407	0.000257	0.1083	0.0083
3_47957996	0.022903	0.1065	0.0065
3_48040284	0.006948	0.1051	0.0083

**Table 2:** True-positive variants in gene MAP4 ordered by MPPs for association with SBP

Variant	MAF	Averaged MPPs	Empirical MPPs
3_48040283	0.028049	0.6059	0.0140
3_47956424	0.343541	0.4082	0.0121
3_47957996	0.022903	0.4065	0.0050
3_47908815	0.000257	0.2553	0.0159
3_47912407	0.002573	0.2083	0.0100
3_47913606	0.000257	0.1485	0.0585
3_48016938	0.000257	0.1310	0.0410
3_47912736	0.000540	0.1106	0.0103

*SNP* single nucleotide polymorphism

## Discussion and conclusions

In this paper, I have applied the Bayesian variable selection approach to study the association between increases in baseline SBP and DBP and genetic variants in the data from GAW19. Although most of the statistical methods designed for rare variant association tests can perform global tests for the association between the region and phenotypes as a result of the low frequencies of rare variants, my method enables detection of not only rare variants, but also of common variants for their significance. My method is highly flexible and allows for uncertainty in estimating parameters in variant selection using the Bayesian framework. The key to my approach is the use of a risk index score and indicator parameters to detect the variant-specific signals. The posterior distributions of all parameters of interest are estimated via MCMC efficiently. I also implemented computationally advantageous permutation within MCMC to calculate empirical thresholds to determine true-positive variants. The detection of disease-associated variants was not sensitive to the MAF thresholds defining rare variants. The application to the GAW19 data reveals that that some common variants and rare variants in the *MAP4* gene are associated with DBP and SBP.

In the application, I have focused on a binary case of inclusion/exclusion of variants. However, my method can be extended to investigate mixed effects (the presence of both protective and risk effects) within the group of rare variants by further assuming *γ*_*j*_ *= −1* if a variant is a risk factor and *γ*_*j*_ *= 1* if a protective factor as in Quintana et al. [16]. This extension may be used to uncover the direction of effects of the variants, but at the cost of substantially increased computation time of the algorithm.

Finally, the current study focused on the specific gene regions. I anticipate that the computational challenges of my method will grow substantially for genome*-*wide searching of rare genetic variants. However, I believe that the qualitative advantages of my approach make it worth investing effort into designing more efficient MCMC algorithms, so as to be able to better deal with very large-scale applications. I leave this to future work.
